# Sustaining physical activity following participation in a joint pain exercise and education rehabilitation programme; a qualitative longitudinal study

**DOI:** 10.3389/fpsyg.2026.1807688

**Published:** 2026-08-17

**Authors:** Panayiotis Michael, Sheree A. McCormick, Jenny Alexanders, Aidan Quinn Innes, Amy Elizabeth Harwood, Francis Fatoye, Patrick Doherty, Nigel Timothy Cable, Davina Deniszczyc, Gillian Yeowell

**Affiliations:** 1 Faculty of Health and Education, Manchester Metropolitan University, Manchester, United Kingdom; 2 Nuffield Health, Surrey, United Kingdom; 3 Faculty of Science and Engineering, Manchester Metropolitan University, Manchester, United Kingdom; 4 Institute of Sport, Manchester Metropolitan University, Manchester, United Kingdom; 5 Department of Health Sciences, University of York, York, United Kingdom

**Keywords:** behavior change, community-based rehabilitation, exercise rehabilitation, muskuloskeletal health, socio-ecological model (SEM), sustained physical activity, transtheoretic model

## Abstract

**Background:**

Musculoskeletal conditions are leading contributors to global disability and place a significant economic burden on health systems. Optimal physical activity is a key recommended strategy for managing musculoskeletal conditions, yet many individuals struggle to sustain activity levels following the completion of exercise rehabilitation programmes. Identifying and understanding the factors that influence sustained physical activity may aid intervention design and benefit future patients. The aim of the study was therefore to explore the evolving needs of participants completing a joint pain exercise rehabilitation programme and identify factors which influenced sustained physical activity beyond programme completion.

**Method:**

The current study adopted a longitudinal semi-structured interview methodology, building upon our initial study conducted at the end of the supported phase (week 12). Semi-structured interviews were conducted at week 24 (mid-term follow up), and at week 36 (long-term follow-up), during which participants were expected to self-initiate engagement with structured physical activity. Reflexive thematic analysis was employed, and interpretation of findings was guided by the Socio-Ecological Model and Transtheoretical Model, to examine changes in behavior and context over time. Participants were classified as either sustainers or non-sustainers based on their overall behavioral trajectory.

**Results:**

Five overarching themes were identified: (1) From avoidance to approach; (2) Becoming an independent exerciser; (3) Establishing a new routine; (4) Outsourcing willpower; (5) Evidence of self-determined behavior. Individual-level factors such as confidence, motivation, and acceptance of pain were central to sustained engagement, but these were shaped by broader influences including access to facilities and social support.

**Conclusion:**

Sustained physical activity following rehabilitation is a dynamic, context-dependent process. Interventions should prepare individuals for independent physical activity by fostering the development of psychological and self-regulatory skills, whilst also considering community and policy-level influences. Incorporating these elements may enhance sustained engagement in structured physical activity and improve long-term rehabilitation outcomes.

## 1 Introduction

Musculoskeletal (MSK) conditions such as joint pain and osteoarthritis affect approximately 1.71 billion people globally, making them the leading contributor to disability (World Health Organisation [WHO], 2022). Prescriptions related to MSK conditions in England alone incur an annual cost of approximately £139 million (Versus Arthritis, 2024). Physical activity (PA) – specifically exercise, a structured and purposeful form of activity – is recommended as an effective strategy for preventing and managing joint pain and other musculoskeletal conditions (Bernstein et al., 2017; Dasso, 2019; De la Corte-Rodriguez et al., 2024; National Institute for Health and Care Excellence, 2022). However, a large proportion of the UK population, estimated between 40% and 75% of adults, are not meeting the UK Chief Medical Officer’s recommended weekly PA levels of at least 150 min of moderate intensity, or 75 min of vigorous intensity activity per week (Department for Health Social Care, 2019; Nuffield Health, 2024; Sport England, 2025). Moreover, an age-related decline in PA has been reported (NHS England, 2024), and many people find it challenging to sustain PA levels once a formal exercise programme has ended (Sansano-Nadal et al., 2019).

Sustained PA is shaped by a complex interplay of determinants, with evidence indicating that age-related decline may be more closely linked to socio-contextual factors, such as familial circumstances and occupational status, than to chronological ageing alone (Klein and Becker, 2011). Prior evidence by Wycherley, Mohr (Wycherley et al., 2012) suggests that continued access to exercise opportunities, including affordable gym memberships, and support via a personal trainer could potentially enhance sustained PA. Additionally, the importance of physical strength and fitness at the end of a supervised intervention, and the individuals’ existing or developed social networks, have also been highlighted as factors likely to predict sustained PA, specifically in older adults (Kendrick et al., 2018). Maula, LaFond (Maula et al., 2019) corroborate the importance of social networks in supporting continued participation post-programme, for example, by engaging in exercise classes with a peer. Psychological determinants of sustained PA identified in prior research include beliefs around the benefits of PA (i.e., identifying physical or mental health improvements), self-efficacy, and the ability to maintain motivation (Amireault et al., 2013; Maula et al., 2019). This highlights the complexity of sustained PA and the importance of considering individual and environmental level factors.

The Socio-Ecological Model (SEM) of health, first introduced by McLeroy, Bibeau (McLeroy et al., 1988), depicts the various interrelationships between individuals and the multiple levels of their surrounding environment that shape their health-related behaviors (Golden et al., 2015). However, the SEM does not capture the underlying processes through which these influences operate, or account for the mechanisms driving change (Joseph et al., 2016; McLeroy et al., 1988). Furthermore, the SEM fails to incorporate the temporal aspects of behavior change, limiting its utility as a standalone theoretical framework for examining sustained behavior change (Hagger et al., 2020; Joseph et al., 2016). The Transtheoretical Model (TTM) addresses this limitation by conceptualizing behavior change as a dynamic process occurring across distinct stages which fluctuate throughout time, each characterized by specific psychological and behavioral requirements at the individual level (Prochaska and Prochaska, 2019; Prochaska and Velicer, 1997; Prochaska et al., 1994). Despite the TTM also facing criticism (Brug et al., 2005; Walton et al., 2022), its prior utilization alongside the SEM (MacCosham et al., 2019), aligns with several authors advocating for tailored frameworks that integrate multiple constructs for a broader conceptualization (Alshagrawi and Alsayed, 2025; Dsouza et al., 2021).

This study constitutes the second and final stage of a two-part longitudinal investigation, employing the SEM and TTM in tandem to identify multi-level factors influencing sustained PA among individuals living with MSK conditions, accessing community-based exercise rehabilitation (McCormick S. A. et al., 2025). In the first study, we reported determinants to the adoption and adherence of exercise rehabilitation, for people accessing a free-of-charge Joint Pain Programme (JPP) delivered by the Nuffield Health charity (Charity Numbers: England and Wales 205533; Scotland SC041793) (McCormick S. A. et al., 2025). The JPP consists of two distinct phases: an instructor-led phase (weeks 1–12) followed by an independent phase (weeks 13–24). During the instructor-led phase, participants attend two weekly sessions, each lasting 60 min (40 min of exercise; 20 min of education), delivered by a Rehabilitation Specialist (upskilled Personal Trainer). The exercise component of the JPP delivered in the first weekly session primarily emphasizes functional movement patterns in a circuits-based format; whilst the second session introduces a variety of exercise modalities (e.g., mobility, stability, strength, cardiovascular) implemented in a format determined by the Rehabilitation Specialist. The educational content of the programme consisted of structured discussions, covering topics such as the importance of exercise for managing joint pain, perceptions and understanding of pain, and goal setting and planning for continued PA. These sessions were intended to support participants in identifying factors that may facilitate or hinder their engagement in PA. A detailed overview of the weekly content, including associated mechanisms of action and targeted behaviors, is provided in Supplementary File 1. Participants are also provided with complimentary access to a Nuffield Health Fitness & Wellbeing Centre throughout both phases, however, the independent phase presents a greater opportunity for participants to utilize this access, as it coincides with the transition away from instructor-led sessions and toward self-initiated engagement with structured PA. Although related work exists in other populations and settings (Gavin et al., 2025; Hartley and Yeowell, 2015; Kendrick et al., 2018; Maula et al., 2019; Wycherley et al., 2012), to the authors’ knowledge, no research has explored factors influencing sustained PA in an MSK population following participation in a community-based exercise rehabilitation programme. Additionally, many studies focus on capturing participant experiences at a single time point (e.g.,13, 14, 32), however, ecological processes (i.e., the ways in which the interactions between the individual and their environment influence behavior) require varying durations to unfold and produce observable change, particularly within complex systems such as community-based rehabilitation programmes (Golden et al., 2015; Hawe et al., 2009). Accordingly, a multi-timepoint longitudinal enquiry was deemed essential to capture participants’ experiences of sustaining PA, and any changes to these, following completion of the JPP (Hawe et al., 2009; Meyers and Davidson, 2025). Research in this area has the potential to improve the economic return of MSK-related research, which received approximately £3.5 billion in funding between 1970 and 2013, by identifying methods to maximize the net monetary benefit through sustained behavior change in this population (Glover et al., 2018). The aim of the study was to therefore explore the evolving needs of participants completing a JPP and identify factors which supported or hindered sustained PA beyond programme completion.

## 2 Materials and methods

### 2.1 Study design

This qualitative longitudinal semi-structured interview study was undertaken to investigate the factors which facilitated or impeded sustained PA in participants completing a JPP. Ethical approval was obtained from Manchester Metropolitan University Faculty Ethics Committee, UK (Ref: 59059). Participants previously interviewed at the end of the instructor-led phase of the JPP (week 12, timepoint 1) (McCormick S. A. et al., 2025), were interviewed again at week 24 (timepoint 2), coinciding with the end of the independent phase. This interview aimed to understand participants’ experience of transitioning to self-initiated engagement with structured PA whilst provided with an ongoing opportunity to engage through the complimentary access to a Nuffield Health Fitness & Wellbeing Centre. A third round of interviews (timepoint 3) was conducted at week 36 to examine the longer-term impact of both JPP phases on overall wellbeing and the maintenance of PA levels, particularly following the conclusion of complimentary access to a Nuffield Health Fitness & Wellbeing Centre. Longitudinal qualitative studies enable “temporal triangulation,” whereby the participants’ narratives are examined across multiple timepoints to identify continuity and changes in their lived experiences (Meyers and Davidson, 2025). Methodological rigor and transparency in the reporting of the research was ensured by following the Consolidated Criteria for Reporting Qualitative Research (COREQ) checklist (Tong et al., 2007).

An inductive methodological approach, grounded within a pragmatic paradigm, was employed to explore participants’ experiences and behaviors following completion of the Nuffield Health JPP. Pragmatism recognizes diverse subjective realities, while concurrently focusing on their practical implications and consequences (Allemang et al., 2022). Given the applied focus of this study (centered on enhancing sustained PA), a pragmatic paradigm offers an appropriate foundation for exploring the dynamic interplay between ecological contexts and PA behaviors (Gillespie and Glaveanu, 2024; Goldkuhl, 2012). Transactional realism, an epistemological approach within pragmatism, emphasizes the ongoing interaction between individuals and their environment, which aligns with the study’s aim of exploring how ecological conditions (including time) shape, and are shaped by, sustained PA (Allemang et al., 2022; Hilderbrand, 2008).

### 2.2 Study population

Initial recruitment for timepoint 1 interviews took place between 8th January 2024 and 2nd February 2024, whereby eligibility was defined as individuals who had recently completed the JPP across five Nuffield Health Fitness & Wellbeing Centers in England, UK, as reported in our previous paper (McCormick S. A. et al., 2025). The present study (timepoints 2 and 3) builds on this work by conducting a longitudinal follow-up of the cohort recruited at timepoint 1. Participants who completed the initial interviews were invited to take part in follow-up longitudinal interviews, ensuring they had relevant experience of attempting to sustain PA following programme completion. Potential research participants expressed interest in the study by contacting a member of the research team (SM), who subsequently provided a participant information sheet and obtained written or oral informed consent from those wishing to proceed. Although the eligible sample was defined purposively based on programme completion and prior research participation, inclusion in the study depended on voluntary self-selection. A total of 21 participants were recruited for the initial interviews (timepoint 1, week 12) and subsequently invited to attend the follow up interviews (timepoint 2 and 3, weeks 24 and 36 respectively) which took place between 15th April 2024 and 14th August 2024.

### 2.3 Data collection

Semi-structured interviews were conducted by SM, an experienced female qualitative researcher. All interviews were conducted remotely, either via Microsoft Teams (video) or telephone. The questions comprising the semi-structured interview guides were collaboratively developed by all authors and informed by the research team’s expertise and the Theoretical Domains Framework (TDF) (Atkins et al., 2017; Supplementary File 2). The TDF domains facilitated capturing key behavioral determinants in relation to sustained PA, whilst the semi-structured format allowed participants to introduce experiences and perspectives beyond those explored in the interview guide, with subsequent questions tailored to their responses. This flexibility enabled the exploration of new and unanticipated issues, supporting an open and inductive approach to data generation. Interviews were digitally recorded and lasted approximately 40 min. All interview data were stored on a password-protected Manchester Metropolitan University server. Recordings were transcribed verbatim by a third-party transcription service bound by a data-sharing agreement with the University, ensuring that all data were handled securely and in compliance with confidentiality requirements. Returned transcripts were pseudonymized by PM, with the original recordings deleted. To recognize their valued contribution to the research, participants received a £10 gift voucher for participating in each interview (up to £30 in total).

### 2.4 Data analysis

All interviews were uploaded into NVivo 14 which was used to organize and code the data. The interviews were analyzed using reflexive thematic analysis, with an initial inductive approach to coding followed by a theoretically informed interpretative phase, in which behavioral frameworks (SEM, TTM) were used to interpret the findings. Transcripts were initially coded by SM, PM and JA, each offering a different positionality in line with their individual experiences and expertise in cognitive psychology, behavioral science and physiotherapy respectively (Braun and Clarke, 2021). The analysis adhered to Braun and Clarke’s six steps of thematic analysis, which provides a rigorous and transparent process that enables researchers to generate patterns of meaning that can be applied across a range of theoretical frameworks (Ahmed et al., 2025; Braun and Clarke, 2021). Thematic analysis is particularly appropriate for healthcare research, where patient experiences are influenced by a complex interplay of emotional, social, and biomedical dimensions (Braun and Clarke, 2014). Reflexive thematic analysis emphasizes the subjectivity of researchers in the interpretation of the data, seen not as a limitation to this methodological approach, but a strength in acknowledging their effort and active role throughout the analytical process (Braun and Clarke, 2019). Consistent with this approach, the research team actively reflected on how their disciplinary backgrounds shaped the interpretive and analytical process. The behavioral science perspective informed attention to behavior change processes, including self-regulation, habit formation, and the intention-behavior gap, influencing how patterns of sustained and non-sustained PA were interpreted. The cognitive psychology perspective brought focus to participants’ beliefs, perceptions, and meaning-making processes. In contrast, the physiotherapy perspective contributed a clinical understanding of movement, pain, and rehabilitation, shaping interpretation of participants’ physical capabilities, exercise behaviors, and responses to symptoms. Differences in interpretation were discussed throughout the analytic process, with multiple perspectives used to deepen understanding rather than to reach a single consensus view. This reflexive engagement enabled a more nuanced and contextually grounded interpretation of participants’ experiences.

Guided by our philosophical stance within pragmatism, a combination of process and descriptive coding was utilized to capture actions, sequences of activity, and outcomes, by assigning codes where possible using terms that reflect ongoing processes e.g., “questioning”; “exercising” (Saldana, 2021). In line with Braun and Clarke’s steps to thematic analysis, transcripts were read and re-read to ensure familiarization with the data prior to coding (Braun and Clarke, 2021). Codes were examined and grouped into preliminary categories which depicted shared meaning and patterns across participants. Relationships between categories were also explored to support interpretation of the processes through which PA behavior was sustained or hindered. The categories were further grouped into the overarching themes. Themes were identified by examining patterns across categories and integrating participants’ accounts from both interview timepoints. The analytical process was iterative and involved regular discussion among the research team, with themes reviewed and refined in relation to both the coded data and the full dataset prior to final agreement. Final themes were defined and named to capture the identified pattern of meaning within the dataset. This approach ensured that themes were generated inductively from the data and are presented in the Results section. Themes were subsequently interpreted using constructs from the TTM, including processes of change, and considered in relation to levels of the SEM to support multi-level interpretation of factors influencing sustained PA behavior. Interpretation of these themes, informed by the behavioral frameworks, is presented in the Discussion.

The process coding also facilitated mapping participants at each interview onto the stages of change proposed within the TTM (i.e., Pre-contemplation, Contemplation, Preparation, Action, Maintenance, Termination) (Prochaska and Prochaska, 2019), using the definitions presented by Pennington (Pennington, 2021). However, given the dynamic nature of change and possibility that participants may or may not sustain their levels of PA, the research team employed alternative descriptors: “sustainers” and “non-sustainers.” These terms more accurately captured the overall behavioral trajectories observed in participants during interviews conducted at weeks 24 (timepoint 2) and 36 (timepoint 3). The Action stage served as a central reference point for assessing post-programme engagement. Participants were classified as sustainers if they were identified as being in the Action or Maintenance stage in relation to their structured PA behavior, with the aim of maintaining or improving their health outcomes. In contrast, individuals assigned to the non-sustainer group were those whose last known stage of change was either Contemplation or Preparation, indicating a return to an earlier stage following completion of the JPP. These classifications were made by the research team through interpretation of the participants’ reported experiences and behaviors within the interview data, rather than through self-classification. Specifically, PM and JA undertook the stage allocation by reviewing the transcripts in accordance with the TTM definitions (Pennington, 2021), with SM acting as a third reviewer in cases of disagreement regarding stage classification. In instances of ambiguity in participants’ accounts, decisions were reached through team discussion, drawing on the overall consistency and direction of participants’ narratives. Example quotes were used throughout this process to support and justify stage allocation during analytic discussions.

Participants were classified based on their most recent observed stage of change, reflecting their latest available behavioral status. For participants who completed the final follow-up interview (week 36, timepoint 3), classification was based on their stage at this timepoint. In cases where participants did not attend the week 36 interview, classification was based on their stage at week 24 (timepoint 2), whilst participants who did not attend the week 24 interview but completed the week 36 follow-up were classified according to their week 36 stage. This approach ensured that classifications were consistently derived from the most up-to-date available data for each participant. The approach also enabled the inclusion of participants that attended at least one follow up interview, and supported the identification of factors crucial to sustaining exercise behavior, through comparisons between sustainers and non-sustainers.

The analysis primarily focused on identifying patterns across participants (between-case comparison) by comparing sustainers and non-sustainers, while also examining narratives across timepoints to capture how new or evolving factors influenced sustained PA over time. As such, themes were primarily structured through comparisons between sustainers and non-sustainers, with temporal data used to explore variation and development within these patterns rather than to define separate timepoint-specific themes.

Methodological rigor and trustworthiness were enhanced through peer debriefing and member checking (Ahmed, 2024). The research team regularly discussed generated codes, resolving divergent interpretation through team deliberation, which embraced a multiple-lens approach rather than seeking a single fixed truth (Rankl et al., 2021). A summary of the findings was shared with participants to ensure their experiences were accurately represented, whilst also adding a further layer of interpretation to the identified themes (Supplementary File 3; Birt et al., 2016; McKim, 2023). This process also empowered participants, fostering a collaborative and participatory approach to the research (Brear, 2019; McKim, 2023). No incentivisation or further reimbursement was provided to participants for their feedback.

## 3 Results

At week 24 (timepoint 2), from the 21 participants invited, 17 participants took part in the interviews. Among the four participants who did not participate, one male participant did not wish to engage in the present study following their week 12 interview (timepoint 1), and three female participants were unavailable due to personal commitments. At week 36 (timepoint 3), 18 participants were interviewed; of the two who did not take part, one male was unable to participate due to ill health, and one female participant did not respond (Table 1).

**TABLE 1:** Summary of participant characteristics and interview attendance.

ID	Gender	Age (Years)	Ethnicity	Interview at 12 weeks	Interview at 24 weeks	Interview at 36 weeks	Sum of change
P1	Female	65+	White British	✓	✓	✓	Sustainer
P2	Female	65+	White Other	✓	✓	✓	Sustainer
P3	Male	65+	White British	✓	Did not wish to participate in longitudinal study		N/A
P4	Male	65+	White British	✓	✓	✓	Non-sustainer
P5	Male	65+	White British	✓	✓	Unable to attend due to ill health	Non-sustainer
P6	Female	65+	White British	✓	✓	✓	Sustainer
P7	Female	55–64	White British	✓	✓	✓	Non-sustainer
P8	Female	65+	White British	✓	✓	✓	Sustainer
P9	Female	65+	White British	✓	✓	✓	Sustainer
P10	Female	65+	White British	✓	✓	✓	Sustainer
P11	Female	65+	White British	✓	✓	✓	Sustainer
P12	Female	65+	White British	✓	✓	✓	Sustainer
P13	Female	65+	White Irish	✓	Unable to attend due to personal commitments	✓	Sustainer
P14	Female	65+	Indian	✓	✓	✓	Non-sustainer
P15	Female	45–54	British/Asian	✓	✓	No response	Non-sustainer
P16	Female	65+	British/South Korean	✓	Unable to attend due to personal commitments	✓	Sustainer
P17	Female	65+	British	✓	✓	✓	Sustainer
P18	Female	65+	White British	✓	Unable to attend due to personal commitments	✓	Sustainer
P19	Female	55–64	White British	✓	✓	✓	Sustainer
P20	Male	65+	White British	✓	✓	✓	Sustainer
P21	Female	65+	White British	✓	✓	✓	Sustainer

Across the two follow-up timepoints (weeks 24 and 36), participants demonstrated distinct overall behavioral trajectories, reflected in their classification as sustainers or non-sustainers. Participants classified as sustainers reported continued engagement in structured PA across the follow-up period, whereas non-sustainers described disengagement from PA over time. Among participants who reached the Action or Maintenance stage at week 24 (timepoint 2), only one described a regression to earlier stages of the TTM at the later follow-up (week 36, timepoint 3).

Five overarching themes were identified through an iterative process of coding, grouping related concepts, and refining patterns of meaning across participants, as described in the Data Analysis section: (1) From avoidance to approach; (2) Becoming an independent exerciser; (3) Establishing a new routine; (4) Outsourcing willpower; (5) Evidence of self-determined behavior.

Data saturation was assessed retrospectively to evaluate whether our findings were sufficiently comprehensive to address the research question, rather than as a criterion for determining sample size or guiding the cessation of interviews (Varpio et al., 2017). Specifically, we assessed the meaning saturation of the initial coding pattern developed i.e., the extent to which all dimensions related to the interpretation of higher-order codes had been captured (Hennink et al., 2017). We examined the point at which no new dimensions, nuances, or insights were identified within these codes across successive interviews. This included consideration of any refinements or additional insights emerging at the later follow-up (week 36), ensuring that saturation reflected a comprehensive and richly developed understanding of the phenomena across the full longitudinal dataset, rather than the mere identification of themes. This approach ensured that saturation accounted for temporal depth, capturing both the stability and refinement of meaning across timepoints.

**FIGURE 1:**
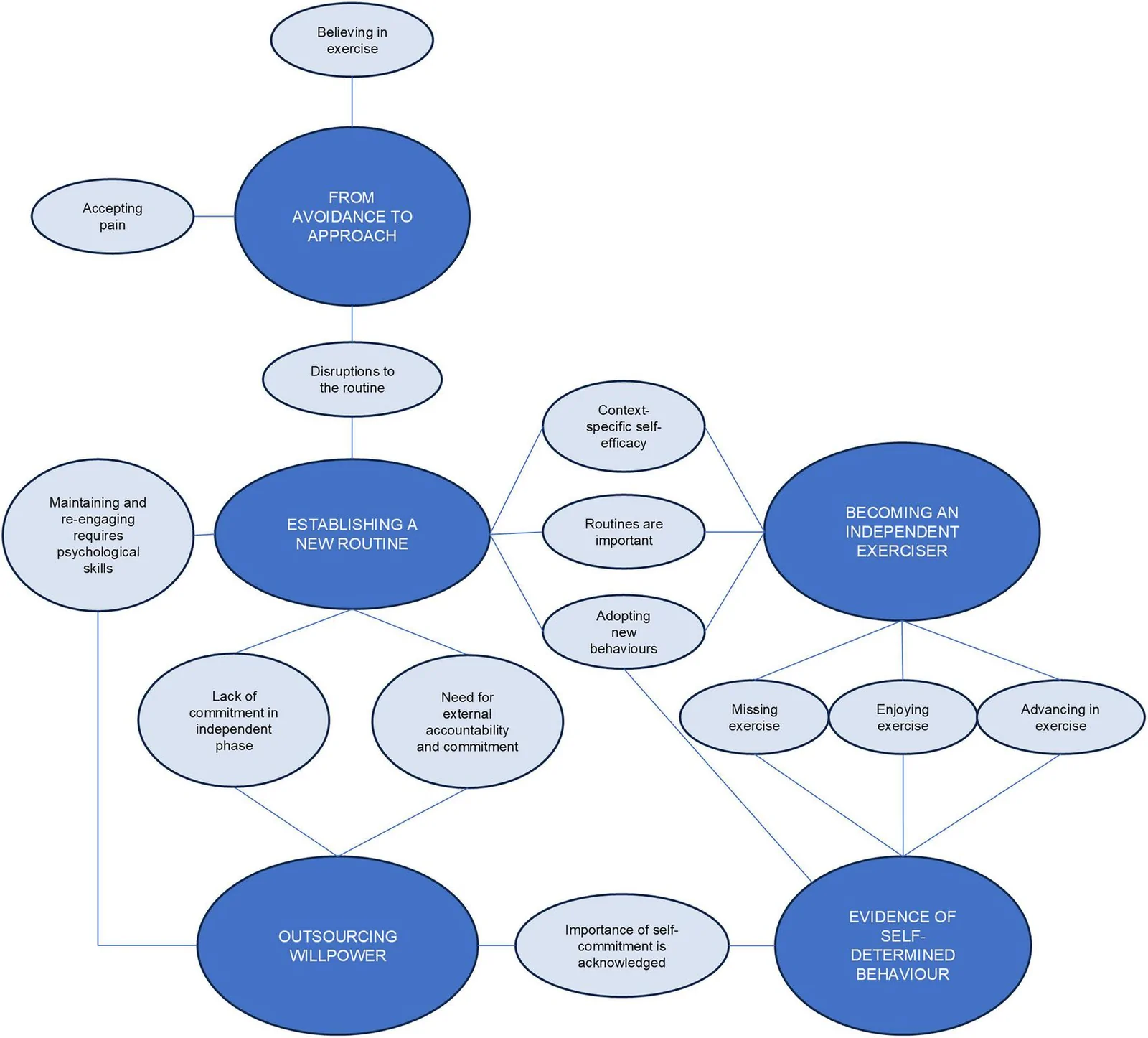
Hierarchy and relationship between themes and sub-themes.

Meaning saturation was reached for all higher order codes following 11 interviews. This was substantiated through triangulation, whereby multiple researcher perspectives were employed to interpret and validate the findings (Fusch and Ness, 2015). The thematic relationships are depicted in Figure 1, where the hierarchy and interconnections are conveyed through the typography of text and color of each shape (ALL CAPS and dark blue indicating overarching themes). The relationships depicted between themes and sub-themes reflect patterns of meaning identified across the dataset, where participants’ accounts highlighted relationships between different factors, illustrating how these influences were experienced as interconnected within the participants’ narratives.

### 3.1 Theme 1: From avoidance to approach

Participants referred to pain as a primary symptom which has persisted throughout their rehabilitation journey. However, participants explained that the pain often fluctuates in its intensity throughout time.

*Yeah, well I’ve got the pain all the time, but it just goes up from… I would say it averages around a three or four for me and then it can go up to nine so it can go quite high.* [P1, Sustainer, Week 24]

*I have something between slight pain and discomfort and moderate pain and discomfort. And that’s to do with the arthritis in my feet.* [P4, Non-sustainer, Week 24]

Across the follow-up period, a key distinction for participants who sustained their structured PA, was their ability to accept pain, acknowledge its fluctuating nature, and manage engagement with exercise accordingly.

*I think it’s something that I’ve got to, sort of, live with, and learn to manage really. I wouldn’t say it’s debilitating pain, but I am aware of it.* [P21, Sustainer, Week 36]

*And I think I can accept it when it’s there, but I treat it differently. I can’t explain it. I just think, “Well, okay, but this won’t last. It will go.”* [P12, Sustainer, Week 24]

*[…] it was made obvious in the group, the fact that you don’t have to just rest up all the time if you’ve done something, and now, I don’t, if I’ve hurt my knee, I don’t rest up, I carry on.* [P18, Sustainer, Week 36]

It was also evident that participants were developing an increased understanding of the relationship between improvements in pain and structured PA, recognizing that relapses would lead to stagnating results, whereas consistent engagement would contribute to reductions in pain.

*I think when I was actually doing the gym, it was helping. I think I was improving. But now because I haven’t been going, I find that nothing’s improved after that, you know?* [P14, Non-sustainer, Week 36]

*But I still have quite a lot of pain in my legs, but because I’ve stepped the exercise up that seems to be helping.* [P9, Sustainer, Week 36]

*My spine was so stiff. So you know, I did a few exercises then before I got out of bed this morning*. [P13, Sustainer, Week 36]

Additionally, several participants adopted monitoring systems, utilizing apps, smartwatches, and other tools to track their daily PA levels. This was more commonly reported by sustainers compared to non-sustainers, including the ability to monitor how levels of PA influenced their pain, further reinforcing their beliefs around the relationship between PA and pain.

*It’s a Garmin. It’s an old one now but that, together with everything, and I’ve got the app on my phone and all of that kind of thing, it just helps really, me motivated to going.* [P16, Sustainer, Week 36]

*I’m trying to do 5,000 to 6,000. If I get to 13,000 steps, my knee feels it, and I know I’ve done too much.* [P1, Sustainer, Week 24]

### 3.2 Theme 2: Becoming an independent exerciser

The shift to self-initiated engagement in structured PA during the transition from the supported to independent phase of the programme (weeks 12–24), was accompanied by participants’ uncertainty and diminished confidence in executing exercise movements correctly without guidance or demonstration.

*I got a programme and I went in and did it once and I had other pain, not pain from muscles working, but joint pains after. So, I think possibly I wasn’t doing it quite right. I don’t know. But I find at least with the classes, I come out… I know I’m doing it right because there’s someone there, and I come out feeling better, so I tend not to go on the gym stuff.* [P10, Sustainer, Week 24]

This was coupled by a hesitancy to engage with or attend classes led by other exercise professionals, with participants often demonstrating a strong dependence on the Rehabilitation Specialist who delivered their JPP sessions during the initial 12 weeks.

*I only like going to [Rehabilitation Specialist]’s class. I’m not interested in going to any other classes that the gym offer at this stage.* [P8, Sustainer, Week 24]

Transitioning into independent PA also required the adoption of new behaviors for many participants. This included learning how to navigate the class booking system and developing the ability to self-regulate exercise intensity within gym and class settings.

*Well, I’d like to get into the classes, but I haven’t really done any as yet. So it’s just getting organized. I was a bit slow at downloading the app. I hadn’t done it when we spoke, for booking in ‘cause it’s a new system to book in.* [P6, Sustainer, Week 24]

*The last time I went to the gym I did a class and there was a chap running it, he really, really pushed us more than I think my knees needed.* [P11, Sustainer, Week 24]

### 3.3 Theme 3: Establishing a new routine

Participants spoke about the importance of establishing a new routine during the transition from the supported to independent phase of the JPP (weeks 12–24), as the structure previously facilitating their routine was no longer present.

*You know you’re going twice a week, you’re committed to going twice a week, and you’re focused on that. When that’s gone, it’s like sort of… I won’t say like a safety net, but the prompt to go has gone, and you’re then having to be independently prompting yourself.* [P1, Sustainer, Week 24]

However, participants that were successful in establishing a new routine, acknowledged its fragility, reporting that PA was a habit that could easily be lost or disrupted.

*I think once you fall out of the habit, it’s really hard to pick it up again. So I just try and keep it going. It doesn’t have to be three or four classes a week. If I could only manage one or two, that’s better than nothing* [P9, Sustainer, Week 24]

Participants discussed a range of physical, social, and psychological factors that could potentially disrupt their routine. These disruptions were particularly evident among non-sustainers, who were more likely to experience a breakdown in routine and a cessation of PA as a result of these challenges. In contrast, sustainers were able to overcome or adapt to these challenges and return to structured PA.

*Not very good, to be honest. I’ve had a few sort of bouts of illness and I think it’s really knocked me back. So unfortunately I’ve not really kept up with the exercise, sadly […] And then I missed a few sessions and wasn’t able to attend. I was going swimming, but I have to admit, that sort of tapered off as well. I’ve still not went, sadly.* [P7, Non-sustainer, Week 36]

*Gradually, they’re still investigating the stomach thing, but I’ve resumed exercise, and I did take up the option to become a member at Nuffield.* [P20, Sustainer, Week 24]

Following the conclusion of the complimentary access to a Nuffield Health Fitness & Wellbeing Centre provided during the JPP (week 24), some participants faced another potential disruptor to their established routine, as not everyone could afford the subsidized membership offered by Nuffield Health. At this stage, participants not only described the immediate impact of this transition but also expressed awareness that these barriers could influence their ability to maintain structured PA beyond the programme. This resulted in further barriers to sustained PA emerging, including the perceived cost-benefit of travelling to a Nuffield Health site, and a lack of confidence in integrating into a new fitness center.

*[…] if it was the full price then it wouldn’t be an option for me. I’m coping with it at the moment. If it goes up, I probably wouldn’t cope with it.* [P19, Sustainer, Week 24]

*I feel like I’m starting again. I do feel like I’m starting again. When we went for an induction at the leisure center, the guy, he didn’t even show us the swimming pool […]* [P15, Non-sustainer, Week 24]

*So I joined the gym round the corner which is a big help because it doesn’t take me as long to get there obviously as it takes to get to the Nuffield one which makes a difference.* [P2, Sustainer, Week 24]

Both sustainers and non-sustainers unwittingly highlighted the role of psychological strategies such as action planning, diarising, reflecting on past successes, and engaging in self-talk, in either supporting the maintenance of PA, or aiding efforts to overcome barriers when attempting to re-engage after a relapse.

Well, immediately, I come back, in my diary actually, I’ve put, “Sign up for the gym. Sign up for a class with [Rehabilitation Specialist].” I’ll be proactive. [P12, Sustainer, Week 24]

*So I might change my exercise and rather it be loadbearing on my feet, the bike ride might benefit me.* [P5, Non-sustainer, Week 24]

*So, the only way that I could get back into this routine now is to literally set an alarm for the morning, which is what some of the other people do. So, I have to set an alarm for the morning that I need to book the course.* [P19, Sustainer, Week 24]

### 3.4 Theme 4: Outsourcing willpower

In support of the above theme, participants reflected on the role of commitment, noting that the transition to the independent phase (weeks 12–24) often involved the loss of an external source of accountability that had previously supported their engagement. The importance of accountability and self-commitment remained evident at the later follow-up (weeks 36, timepoint 3) for both sustainers and non-sustainers.

*Well the programme I suppose you, not have to go but do you see what I mean, you commit to it and you go, don’t you, whereas now I suppose it’s easier not to in a way […]* [P11, Sustainer, Week 24]

Similarly, many participants expressed a sense of dependency on the programme, suggesting that continued support beyond the initial 12 weeks would have been beneficial in maintaining engagement.

*I think if I was for example to say to [Rehabilitation Specialist], my trainer, I could do with another 6 weeks, if somebody drops out, can I just join in, because that position has already been paid for you if you know what I mean, but I would get so much benefit from that.* [P15, Non-sustainer, Week 24]

*[…] we suggested to [Rehabilitation Specialist] that a follow-on class, if you like, rather than a programme would be good.* [P9, Sustainer, Week 24]

Participants expressed the need for external accountability, with some sustainers actively leveraging this and utilizing it to help them maintain their PA behavior.

It was the commitment, making the commitment because I wasn’t making the commitment to me, I was making the commitment to [Personal Trainer]. [P2, Sustainer, Week 24].

Despite this, the role of self-commitment was acknowledged, both as a tool for sustaining PA and for re-engaging after a setback.

*I think it’s just really me. I think I’ve just really got to get up and do it. I think that’s what it boils down to […] I think it just boils down to me. I’ve just got to get up and go.* [P7, Non-sustainer, Week 36]

### 3.5 Theme 5: Evidence of self-determined behavior

Sustainers demonstrated attitudes indicative of intrinsic motives such as enjoyment of PA, which were already evident for many during the transition from the supported phase to the independent phase of the programme (weeks 12–24).

*Oh, it makes me feel good. Particularly more so cycling and being out in the fresh air, but the gym does have a good effect as well.* [P9, Sustainer, Week 24]

Sustainers were also more likely to report feeling a sense of absence when they were unable to engage in PA.

*I think it would be me that… because I would hate myself. I would hate how I felt by not exercising.* [P8, Sustainer, Week 36]

Sustainers expressed a desire to advance their exercise routines, either by increasing intensity or introducing greater variety. However, some conveyed uncertainty regarding the most effective means of achieving these aims, leading a few to seek guidance from personal trainers.

*They don’t tell you what weights you need to use. Obviously, people choose for themselves but I’m nearly there I think to move up an extra kilo as it were*. [P17, Sustainer, Week 36]

*It will be nice to be able to move on to doing something that’s a bit more like weight training based or something.* [P10, Sustainer, Week 36]

*Well, I booked two personal trainer sessions to get a programme together so I’m sort of doing a version of that. So, I have a bit of a legs day and a bit of an arms day* [P2, Sustainer, Week 24]

## 4 Discussion

**FIGURE 2:**
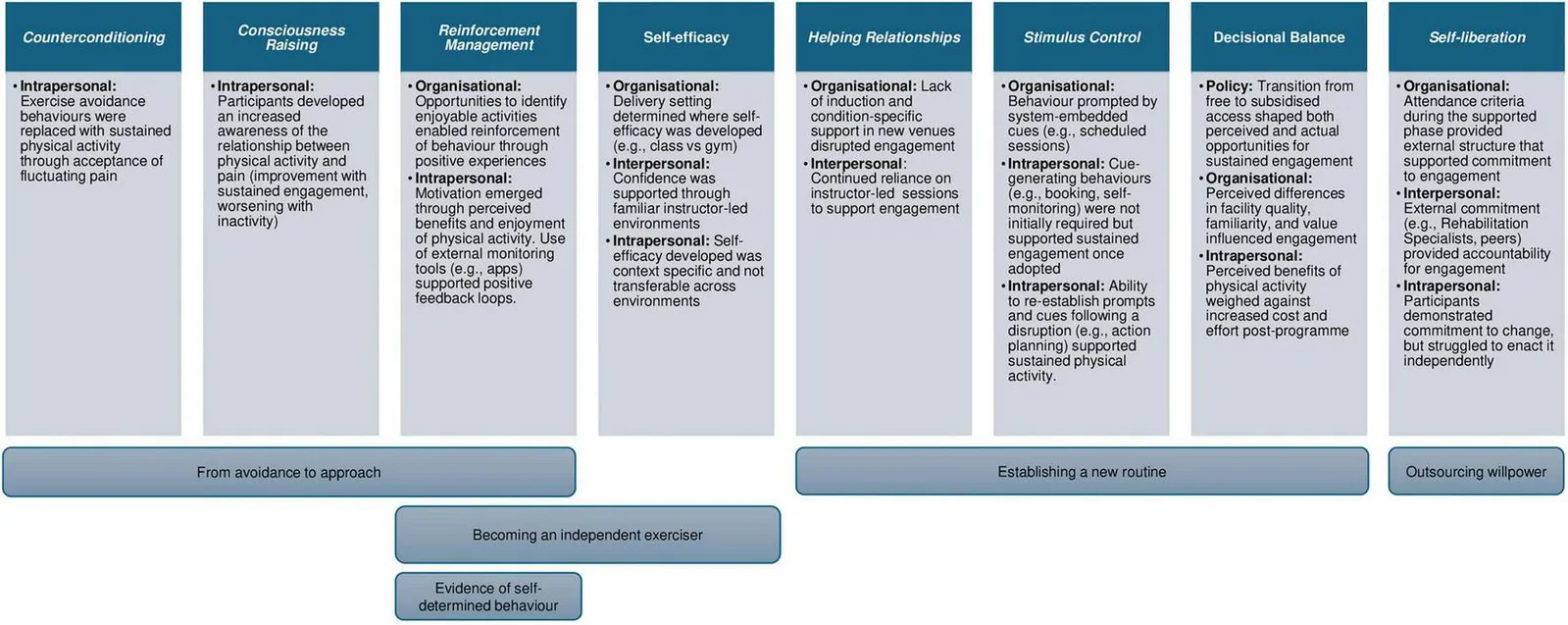
Mapping factors influencing sustained physical activity following the Joint Pain Programme to the Transtheoretical Model and levels of the socio-ecological model (SEM). Italicized terms denote processes of change within the Transtheoretical Model, whereas non-italicized terms represent related constructs. We also indicate the themes to which each mapping corresponds to.

To our knowledge, this study is the first to explore how the evolving ecology of participants who have completed exercise rehabilitation for their joint pain supports or hinders sustained PA. The five overarching themes identified (From avoidance to approach; Becoming an independent exerciser; Establishing a new routine; Outsourcing willpower; Evidence of self-determined behavior) were compared across both sustainers and non-sustainers to identify factors likely to indicate sustained PA. We utilized the TTM to classify participants based on behavioral trajectories (i.e., sustainers and non-sustainers), and building on this, draw on the model to consider how key TTM constructs, including processes of change (italicized throughout), help to interpret the findings presented in the Results and Figure 1 (DiClemente and Graydon, 2020; Prochaska and Prochaska, 2019; Prochaska and Velicer, 1997). These findings are further situated within the SEM to illustrate how determinants of sustained PA operate across various levels of influence (see Figure 2; McLeroy et al., 1988). It is important to acknowledge that the constructs within the TTM (e.g., processes of change) did not emerge in isolation but were likely influenced by the educational and behavioral components of the JPP (e.g., goal setting, activity monitoring, and perceptions of pain; see Supplementary File 1).

As mentioned in the Results, only one participant that reached at least the Action or Maintenance stage at week 24 (timepoint 2) regressed back to pre-contemplation or contemplation at week 36 (timepoint 3). This suggests that behavioral trajectories and the ecological factors influencing sustained PA may be largely established by week 24 of the JPP, highlighting the importance of the transition from supported to independent PA as a critical period for supporting long-term behavior change following community-based exercise rehabilitation.

### 4.1 From avoidance to approach

At the intrapersonal level, the importance of accepting pain for sustained PA became apparent throughout the interviews with participants. Our findings support prior research which has indicated that the acceptance of pain is significantly associated with wellbeing and health outcomes, and no associations previously found between outcomes and actual levels of pain (McCracken and Eccleston, 2005; White et al., 2024). Interventions such as acceptance and commitment therapy have been shown to improve pain interference even when pain intensity remains relatively unchanged (Veehof et al., 2016). Whilst further research is required in the application of brief group-based acceptance and commitment therapy sessions (Majumdar and Morris, 2019), the JPP could consider utilizing such approaches to support participants that may otherwise struggle to accept pain, helping them replace avoidance with active engagement. This is in line with our findings that sustainers recognized that pain did not require cessation of PA and instead described managing symptoms and adapting exercises to maintain engagement, which may be understood as counterconditioning within the TTM, whereby avoidance behaviors (e.g., PA disengagement) are replaced with proactive behaviors (sustained PA).

Underpinning the above, is the process of consciousness raising, through which participants acknowledged the benefits of structured PA on their levels of pain. This is reflected in our findings, where participants described becoming increasingly aware of the relationship between their levels of PA and pain, recognizing that consistent engagement was associated with improvements, whereas periods of inactivity resulted in worsening symptoms. Notably, these patterns of awareness and understanding may have also been shaped by the educational content delivered within the JPP, which explicitly addressed perceptions of pain and the relationship between exercise and symptom management. Therefore, the process of consciousness raising observed in participants’ accounts may reflect the intended impact of these sessions.

Supporting this, the process of reinforcement management was evident in participant’s experiences, with outcomes shaping behavior via a negative feedback loop occurring when reductions in PA levels resulted in a recurrence or intensification of pain, reinforcing continued engagement and acting as a motivational anchor for many sustainers. In line with Maula, LaFond (Maula et al., 2019), beliefs around the benefits of PA appear predictive of sustained PA. These beliefs were further reinforced through external aids such as apps and smartwatches, which were not part of the initial JPP intervention, but adopted independently by most sustainers, as also observed by Maula, LaFond (Maula et al., 2019). Introducing the option of external aids to JPP participants, may support reinforcement management and enhance motivation through positive feedback loops (Carvalho et al., 2025), but also support the avoidance of symptom exacerbation – a key barrier for many participants.

### 4.2 Becoming an independent exerciser

Within this theme, further intrapersonal level factors were identified as crucial to sustained PA. In the first study, we reported that during the first 12 weeks of the JPP, participants experience increases in self-efficacy which coincide with continued participation (McCormick S. A. et al., 2025). During the independent phase, self-efficacy remains an important factor and appears to be a predictor of sustained PA, with our findings corroborating previous research (Maula et al., 2019; Sniehotta et al., 2005; Strachan et al., 2007; Yang et al., 2024). In our findings, participants described feeling confident within familiar, instructor-led environments but less assured when required to engage independently or in new settings. The findings of the present study therefore suggest that self-efficacy may not be universally transferable, rather, the context in which it is developed, and ability to perform the behavior under the same conditions, serves as a critical determinant of an individuals’ belief about their capabilities.

The TTM highlights the importance of the interplay between self-liberation and self-efficacy, particularly during the volitional stages of change, where individuals commit to action based on a belief in their ability to change (DiClemente and Graydon, 2020; Prochaska and Velicer, 1997). However, our findings challenge the assumption that self-efficacy is uniformly applicable across various environments for the same behavior, even when self-liberation is still present i.e., there is a commitment to change without the corresponding confidence to enact that change. As such, the JPP may benefit from exposing participants to a variety of PA environments within the exercise modalities included (e.g., studio, gym-based, free-weights, machines), thereby supporting the development of multiple context-specific self-efficacies that are likely to underpin sustained behavioral engagement.

The transition to self-initiated engagement with structured PA also introduced new prerequisite behaviors and intra-activity capabilities that participants must engage with and demonstrate. Firstly, participants are required to identify or create their own PA opportunities. Notably, participants reported a continued reliance on instructor-led sessions, reflecting the process of helping relationships and the context-specific nature of their self-efficacy. However, this required participants to book onto sessions using the existing mobile application, which also served as a prompt to committed action for many (stimulus control). Downloading the app, and using it to book onto sessions, became pre-requisite behaviors to structured PA that were not previously required, as places were automatically secured for participants during the supported phase of the JPP. Similarly, participants were now also required to independently self-regulate their intensity and volume of PA, whether participating in a timetabled session or independently exercising, a behavior not demanded to this extent during the supported phase. Here again the process of self-liberation and self-efficacy appear vital, as participants must possess the commitment and confidence to undertake these new behaviors (DiClemente and Graydon, 2020). Research by Kanning (Kanning, 2010), demonstrated the importance of self-liberation and stimulus control for the maintenance of PA in the post-action stage of coronary artery disease rehabilitation. Our findings support and extend this work by adding contextual insight specific to the JPP. Furthermore, they align with Kanning’s proposal to consider self-regulatory processes not explicitly captured within the TTM (Kanning, 2010). Accordingly, the JPP could benefit from introducing these prerequisite behaviors and intra-activity skills to participants during the supported phase, enabling them to develop the necessary skills required within a supportive environment, and better preparing them for greater autonomy and sustained PA.

### 4.3 Establishing a new routine:

Remaining within the intrapersonal level of influence, participants highlighted the importance of routine across their rehabilitation journey. At any point, new unanticipated ailments, but also existing joint pain, were disruptors to established routines for participants. As noted within our theme “From avoidance to approach,” one’s ability to accept their pain was predictive of sustained PA, and this could be explained by the ability to better avoid such disruptors to their established routines. Our findings also align with the process of stimulus control, whereby routines act as cues and prompts to the target behavior (i.e., sustained PA) (Walton et al., 2022). Participants classified as non-sustainers described disruptions to their routines following new or worsening physical health conditions, alongside difficulty adapting to or overcoming these challenges and returning to previously established routines. This suggests a reduced ability to maintain behavioral cues, which can be interpreted as a more fragile stimulus control. When sustainers experienced reductions in stimulus control, they were more effective in deploying strategies such as diarising and action planning, to help them overcome this reduction and re-engage with structured PA. This is consistent with the JPP’s emphasis on behavioral planning and self-monitoring, including the use of activity diaries and structured discussions around planning for sustained PA, which may have supported the re-deployment of behavioral cues by sustainers. These strategies are recognized behavior change techniques within the behavior change technique taxonomy and ontology e.g., 1.4 Action planning, 15.4 Self-talk and 15.3 Focus on past success (Marques et al., 2023; Michie et al., 2013). The techniques facilitate behavior change through mechanisms of action such as behavioral cueing and increase in one’s belief about their capabilities, or as highlighted within the TTM, stimulus control and self-efficacy (DiClemente and Graydon, 2020; Johnston et al., 2021). Explicitly linking these techniques within the JPP to specific temporal and contextual cues (e.g., following disruptions to routines) may increase their perceived relevance and encourage their use among non-sustainers.

At the organizational level, the complimentary access to a Nuffield Health Fitness & Wellbeing Centre was a motivating factor for initial engagement with the JPP (McCormick S. A. et al., 2025). However, following the conclusion of the complimentary access at week 24, another potential disruptor to the established routine and stimulus control for many participants emerged. At this point, participants described a need to reconsider whether continued engagement was worthwhile, particularly when balancing cost, travel, and perceived value of access. These accounts suggest that multiple interacting factors, including affordability, accessibility, and familiarity of the exercise environment, influenced participants’ decisional balance for sustained PA, which highlight interactions across various levels of the SEM (DiClemente and Graydon, 2020; Prochaska et al., 1994).

Participants that were unable to afford the subsidized membership, or simply favored alternative gyms or fitness centers, referred to the difficulties experienced when attempting to integrate into these new environments. Our findings further illustrate the relevance of context-specific self-efficacy at the intrapersonal level, alongside interpersonal factors such as unfamiliarity with other members and staff, and the loss of previously established helping relationships (McCormick S. A. et al., 2025). Facility infrastructure and available amenities were also evident as significant organizational factors within a complex system of influences on sustained PA. These results support prior research by Wycherley, Mohr (Wycherley et al., 2012), as the authors also identified that continued access to appropriate PA opportunities and affordable gym memberships may facilitate sustained PA behavior. As mentioned in our previous paper (McCormick S. A. et al., 2025), policymakers and intervention commissioners should carefully consider the cost-benefit of lifestyle interventions, including subsidizing gym memberships specifically for individuals with lower resources, and the potential impact this may have on health outcomes and reductions in healthcare costs.

### 4.4 Outsourcing willpower

Research by Kendrick, Orton (Kendrick et al., 2018) and Maula, LaFond (Maula et al., 2019) demonstrated the importance of social networks in maintaining PA in older adults. Our previous work has similarly demonstrated the role of social networks in exercise rehabilitation (McCormick S. et al., 2025; McCormick S. A. et al., 2025). Whilst our findings align with this perspective, particularly regarding the importance of helping relationships, we propose further nuance to this argument, whereby a fundamental mechanism through which intervention-facilitated social networks support sustained PA, is via external accountability and commitment. Almost all participants discussed the need for external commitment, which seemingly, may indicate an interpersonal level determinant to sustained PA. Similarly, participants suggested that organizational factors, such as the continued provision of services (e.g., graduate classes), may have cascading effects across multiple levels of the SEM, helping them to overcome barriers such as low self-efficacy, motivation and commitment. Our findings align with facilitators reported in other healthy lifestyle interventions, where continued service provision and support from personal trainers were identified as key enablers of sustained PA (Wycherley et al., 2012). As above, the cost-benefit of providing organizational or policy support should be considered.

Building on this, the results also suggest a key distinction within self-liberation. While self-liberation is conceptualized as an intrapersonal commitment to change, participants frequently relied on external structures (e.g., instructor-led sessions and scheduled classes) to initiate and sustain engagement. This indicates that the enactment of self-liberation was outsourced or supported by interpersonal and organizational factors, likely reflecting limitations in self-regulatory capacity. This pattern reflects the intention-behavior gap, whereby individuals demonstrate a commitment to change but struggle to translate this intention into action (Bassett, 2015; Rhodes and De Bruijn, 2013). Thus, reliance on external commitment may not represent an interpersonal alternative to self-liberation, but rather highlights a limitation in participants’ ability to enact this process independently. This underscores the importance of self-regulatory and motivational processes in PA maintenance, also proposed in prior work (Amireault et al., 2013; Kanning, 2010). Participants in our study acknowledged the importance of self-commitment, yet described difficulty enacting this independently, further highlighting the need for self-regulation; defined as the cognitive and psychological skills one possesses to help them regulate their behavior toward a desired end goal (Carver and Scheier, 2012). Theories of relapse emphasize self-control as a central component of self-regulation, and a key factor in understanding lapses and relapses in behavior change (Baler and Volkow, 2006; Baumeister, 2003). In the absence of external support, self-control may therefore represent a critical target within health interventions such as the JPP, to support behavioral maintenance and improve health outcomes.

### 4.5 Evidence of self-determined behavior

In contrast to their reliance for external commitment, sustainers did however demonstrate an internalization of motives to engage in PA. Research underpinned by self-determination theory has frequently depicted that a shift from extrinsic motivational states toward more autonomous states (both identified and intrinsic), is predictive of short-term and long-term exercise behavior (Fortier et al., 2012; Teixeira et al., 2012). Our findings further corroborate these prior results, with sustainers reporting a strong enjoyment of exercise, a sense of absence when unable to engage in PA, and the expression of clear goals and values related to exercise. These patterns suggest an internalization of their motivation toward PA (Deci and Ryan, 2008; Ryan and Deci, 2017). In line with the TTM and SEM, this internalization is indicative of reinforcement management at the intrapersonal level, where participants begin to experience a natural reward from being physically active through a sense of enjoyment, and a negative consequence in the feeling of loss when unable to participate. Supporting participants within the JPP to identify exercise opportunities they enjoy could enhance the likelihood of sustained PA.

The commitment to new goals and health values also represents another step toward self-liberation for sustainers, with organizational-level factors, such as the opportunity to pursue these new ambitions, playing a crucial role. Many participants highlighted the need for intermediate-level exercise opportunities, where they can progress from their previous levels during the supported phase of the JPP, but not to a degree that makes participation unfeasible. The concept of “Zone of Proximal Development” introduced by Vygotsky within the Sociocultural Theory of Cognitive Development, could provide a framework for both interpreting and translating these results into meaningful action (Vygotsky, 1980). The concept suggests that there is a unique zone between what an individual can and cannot perform independently, within which they can succeed with the aid of external support, gradually reducing this support to enhance independence (Smagorinsky, 2018). Whilst the framework and corresponding research has predominantly focused in the area of child development and education, more recent applications have also applied it within the public health domain (Vainio et al., 2014; Wilson et al., 2025). Within the context of the JPP, offering intermediate-level graduate classes during the independent phase, and thus exposing participants to more challenging exercises than in the supported phase, could enhance participant self-efficacy, and help fulfil the new ambitions of sustainers.

### 4.6 Practical and policy implications

In considering the practical and policy implications of these findings, it is important to first recognize the contextual factors that may shape their transferability. Whilst this study was conducted within a UK-based, charity-funded rehabilitation programme context, Nuffield Health provided participants with structured support and subsidized access to fitness facilities that may not be present across all healthcare systems or community settings. In contexts with fewer resources, where access to facilities, professional guidance, or continued programme provision may be more limited, the barriers to sustained PA identified in this study may be further exacerbated. Nevertheless, the underlying mechanisms identified within the findings, such as the development of self-efficacy, the establishment of routine, and the role of social and organizational support, are likely to be relevant across a range of contexts. However, their expression and impact may vary depending on local infrastructure, resource availability, and the structure of rehabilitation services. As such, the transferability of these findings lies primarily in the behavioral processes identified, which may require adaptation to align with different community contexts and health systems.

Taken together, our findings highlight several important implications for practice and policy within community-based rehabilitation. At a programme level, the transition from supported to independent PA represents a critical period that requires structured preparation, including the development of self-regulatory skills such as action planning, self-monitoring, and relapse management. At a systems level, sustained engagement was also shaped by access to appropriate environments, with the withdrawal of subsidized facility access emerging as a key disruptor. This highlights the importance of considering affordability, continuity of provision, and service integration within community-based rehabilitation pathways. Interventions should also consider providing opportunities for graded autonomy, whereby participants are gradually supported to select exercises and plan their own sessions, to reduce the abrupt removal of external accountability. More broadly, the findings emphasize that promoting sustained PA requires alignment across individual, organizational, and policy-level factors. Designing rehabilitation pathways that account for these interacting influences may enhance long-term adherence and improve outcomes for individuals living with musculoskeletal conditions.

### 4.7 Strengths and limitations

A key strength of this study is that the research interviewer (SM) was present at all interviews allowing for the development of rapport with participants over time. This prevented participants having to repeatedly recount their story at each timepoint. At the final interview participants were asked if they felt attending the interviews had influenced their participation. Participants reported that they found the interviews beneficial in providing an opportunity for reflection, highlighting the progress (or lack thereof) they had made. We intentionally oversampled beyond the point of data saturation at timepoint 1 (week 12) to mitigate the effects of potential participant attrition, and to ensure sufficient data availability for achieving meaning saturation in the subsequent timepoints (weeks 24 and 36) (McCormick S. A. et al., 2025).

Attrition was, however, less than anticipated and this may have been influenced by the established participant-researcher relationship. On one hand, this highlights the benefit of longitudinal studies in establishing relationships and eliciting rich, meaningful data (Calman et al., 2013). However, it may have introduced potential bias, as participants’ reflections on their progress may have reinforced or cued them to take action. The longitudinal nature of the study provided multiple opportunities for participants to reflect on their behavior and progress. This may have functioned as an unintended behavioral intervention, prompting self-monitoring, goal evaluation, and renewed motivation to engage in PA. While this reflexive engagement may have enriched the data collected, and provided deeper insight into behavioral processes, it is also possible that it contributed to sustained PA among some participants. Future studies may wish to consider alternative designs or incorporate comparison groups alongside objective measures of PA to further explore these effects.

Additionally, participants were self-selected into the study, which may have introduced selection bias, as those who chose to participate may have been more motivated and engaged with PA than the wider programme population. However, it is important to note that the study also included participants who were unable to sustain their PA, and only one participant interviewed at timepoint 1 (week 12) did not take part in a follow up interview. This ensured that barriers to continued engagement were still captured, contributing to a more comprehensive understanding of sustained and non-sustained behavior.

The use of the TDF in the interview guide ensured comprehensive coverage of key behavioral determinants however, it may have influenced how participants framed their responses. The semi-structured format mitigated this by allowing participants to introduce experiences and perspectives beyond the predefined domains, enabling the capture of data that extended beyond the framework.

A further strength of the study is the application of the TTM and SEM in a multi-timepoint longitudinal study, which provided time for ecological process to unfold (Hawe et al., 2009) and enabled the identification of factors which were contextually and time dependent e.g., self-efficacy and commitment; and the cascading effects of multi-level influences on these. Furthermore, our analysis demonstrated that integrating multiple theoretical models and frameworks helps overcome the limitations inherent in relying on a single framework alone (Alshagrawi and Alsayed, 2025; Brug et al., 2005; Dsouza et al., 2021).

When applying the stages of change definitions, we identified a discrepancy between participants’ perceptions of maintenance and the objective definition of this stage. Specifically, participants associated maintenance with sustaining a previously established routine rather than continuous engagement with PA. Some participants were intentional about avoiding relapse but did not meet the goals or expectations they had set for themselves. Consequently, their subjective evaluation of their behavioral trajectory was not one of progress. Relying on participants’ self-reported stages of change rather than our own mapping, may have influenced our analysis and interpretation of the findings (Kahlert, 2015). Moreover, two participants defined as non-sustainers did not attend the final week 36 interview; therefore, it is possible that they re-engaged with structured PA and their subsequent lived experiences, if captured, may have also influenced our interpretation of the data.

Lastly, as the study was conducted within a specific programme context (the Nuffield Health JPP) which included subsidized access and structured support, the transferability of the findings to other settings may be influenced by differences in resource availability and service provision.

## 5 Conclusion

This study offers a novel contribution by capturing the dynamic interplay between ecological and behavioral determinants of sustained PA following exercise rehabilitation, and by demonstrating how these interact longitudinally rather than in isolation. Our findings highlight the need for adaptive, ecologically responsive intervention designs. Intrapersonal determinants such as self-efficacy, motivation and self-regulation are central, however, their development and maintenance can be influenced by organizational support and interpersonal relationships. Our findings suggest that behavior change in an MSK population is not always linear but can be cyclical, shaped by life circumstances such as the onset of new comorbidities or financial status. Interventions aiming to promote sustained PA should adopt a systems-informed approach, embedding opportunities for psychological skill development and ecological continuity throughout the rehabilitation journey. This may include action planning, problem solving, and self-regulatory skill development within the intervention, alongside opportunities for PA that these skills can be applied in. Additionally, it is important for intervention designers to recognize that broader factors, such as community infrastructure and cross-sector collaboration, play a critical role in supporting individuals living with MSK conditions. For example, formal partnerships with local leisure services or opportunities for participants to engage in community-based peer-led PA groups, may support participants during their transition away from supported rehabilitation. The significance of the findings of the present study is that considering and incorporating these elements may enhance long-term engagement in structured PA, reduce relapse, and ultimately contribute to improved health outcomes and reduced healthcare burden associated with MSK conditions.

## Data Availability

The datasets presented in this article are not readily available because full interview transcripts carry a risk of deductive disclosure, and the richness of participants’ narratives may make individuals identifiable even after anonymization. A minimized, anonymized dataset supporting the findings will be made available by the corresponding author upon reasonable request. Requests to access the datasets should be directed to panayiotis.michael@stu.mmu.ac.uk.
