# Supplementary material for: Sustaining physical activity following participation in a joint pain exercise and education rehabilitation programme; a qualitative longitudinal study
Source: Front Psychol. 2026 Aug 17;17:1807688. doi: 10.3389/fpsyg.2026.1807688 (PMC13526682; doi:10.3389/fpsyg.2026.1807688)
Supplement: Supplementary file 1 [file Table_1.docx]

**Education topics within the Joint Pain Programme**

|  | **Day 1** | **Day 2** | **Mechanisms of Action*** | **Target behaviour(s) or outcome** |
| --- | --- | --- | --- | --- |
| **Week 1** | Introduction and goal setting | What is joint pain? | - Goals - Knowledge - Beliefs about consequences | - Exercising - Any other behaviour / outcome determined by participants |
| **Week 2** | Why is exercise important? | Activity and exercise diary | - Knowledge - Attitude towards the behaviour - Beliefs about consequences - Feedback process | - Exercising |
| **Week 3** | Emotional wellbeing | NHS emotional wellbeing advice | - Knowledge - Social influences - Emotions | - Improving emotional wellbeing |
| **Week 4** | Perceptions of pain | Ask yourself questions to thrive | - Knowledge - Belief about consequences - Attitudes towards the behaviour - Behavioural cueing | - Exercising - Recognising differences in pain e.g., Delayed Onset of Muscle Soreness |
| **Week 5** | Healthy eating | Acts of kindness to others | - Knowledge - Memory, attention and decision processes | - Healthy eating (inc. portion control) - Improving emotional wellbeing |
| **Week 6** | Goal checking | Living your values | - Goals - Feedback process - Values | - Exercising - Any other behaviour / outcome determined by participants |
| **Week 7** | Exercise and programming (FITT) | The rule of opposites | - Knowledge - Emotions - Behaviour regulation | - Exercising - Improving emotional wellbeing |
| **Week 8** | Pain management | Be happy that you are happy | - Knowledge - Belief about consequences - Attitudes towards the behaviour - Emotions | - Managing flare ups - Identifying pleasure in life |
| **Week 9** | Weight management | Accept your mood | - Knowledge - Belief about consequences - Emotion | - Managing weight for joint health - Managing low mood or anxiety |
| **Week 10** | Sleep, rest and recovery | Sleeping better | - Knowledge - Belief about consequences - Attitude towards the behaviour - Behavioural cueing | - Improving sleep routine and hygiene |
| **Week 11** | Planning for success | Worry less | - Knowledge - Behavioural cueing - Emotion - Memory, attention and decision processes | - Exercising - Managing worries |
| **Week 12** | Next steps | Rule of the purple cat | - Goals - Emotion | - Exercising - Any other behaviour / outcome determined by participants |

*Note.* Participants are provided with a handbook which allows them to follow the topic discussions and self-monitor their exercise progress.

*Mechanisms of action reported in accordance with the Theory and Techniques Tool developed by Johnston et al. (2021).

JOHNSTON, M., CAREY, R. N., CONNELL BOHLEN, L. E., JOHNSTON, D. W., ROTHMAN, A. J., DE BRUIN, M., KELLY, M. P., GROARKE, H. & MICHIE, S. 2021. Development of an online tool for linking behavior change techniques and mechanisms of action based on triangulation of findings from literature synthesis and expert consensus. *Translational Behavioral Medicine,* 11**,** 1049-1065.
