# Supplementary material for: Sustaining physical activity following participation in a joint pain exercise and education rehabilitation programme; a qualitative longitudinal study
Source: Front Psychol. 2026 Aug 17;17:1807688. doi: 10.3389/fpsyg.2026.1807688 (PMC13526682; doi:10.3389/fpsyg.2026.1807688)
Supplement: Supplementary file 3 [file Table_3.docx]

**SUBJECT:** Your Feedback Is Welcome

Hi,

Thank you once again for taking part in our research exploring experiences of physical activity following the Joint Pain Programme (JPP).

We would like to share with you the main findings (themes) of our project and would be very grateful if could kindly volunteer some feedback about this. Please note that to create a theme, we gathered the points that were made by most of the participants. **If you could offer comments as to whether you generally agree with the findings, or have any queries, your opinion would be very much appreciated. You can do this by replying to this email.**

**1.** **From Avoidance to Approach**

Many participants described a shift in how they related to pain. Rather than avoiding activity due to discomfort, some began to accept pain as part of their condition and found ways to manage it while staying active. Some participants also used tools like step counters or apps to monitor their activity and better understand how movement affected their symptoms.

**2.** **Becoming an Independent Exerciser**

Participants spoke about challenges they faced when transitioning from a structured, supported programme to exercising independently. Some participants felt unsure about doing exercises correctly without supervision, and many expressed a strong preference for the Rehabilitation Specialist they had worked with during the JPP. Navigating the gym environment and using the booking app also created barriers. However, those who adapted described learning to self-regulate and gradually build confidence in new settings.

**3. Establishing a New Routine**

The end of the structured programme meant the loss of a regular schedule for many. Participants who managed to establish a new routine often described it as fragile, easily disrupted by illness, life events, or changes in access to facilities. Those who sustained activity tended to find ways to adapt and return to exercise after setbacks, while others found it difficult to re-engage once their routine was broken. Cost and travel were also factors that influenced whether people could maintain their new habits.

**4. Outsourcing Willpower**

During the JPP, many participants felt a sense of commitment and accountability that helped them stay motivated. After the programme ended, this external support was no longer present, and some found it harder to stay on track. Participants acknowledged the importance of self-commitment and motivation throughout their post-programme journey.

**5.** **Evidence of Self-Determined Behaviour**

Participants who sustained physical activity over time often showed signs of internal motivation. They spoke about setting personal goals, tracking their progress, and feeling a sense of enjoyment from exercising. Many participants who demonstrated this internal motivation also wanted to continue to progress in their exercise type and intensity but felt unsure about how to best achieve this.
